# Hepatitis in children with tuberculosis: a case report and review of the literature

**DOI:** 10.1186/s12890-020-01215-6

**Published:** 2020-06-16

**Authors:** Maria Sanchez-Codez, W. Garret Hunt, Joshua Watson, Asuncion Mejias

**Affiliations:** 1 Puerta del Mar University Hospital, Cadiz, Spain; 2 Division of Pediatric Infectious Diseases, Nationwide Children’s Hospital, Columbus, OH USA; 3 Center for Vaccines and Immunity, Abigail Wexner Research Institute at Nationwide Children’s Hospital and The Ohio State University, Columbus, OH USA

**Keywords:** Liver function tests; hepatic tuberculosis, Hepatitis, Children, Transaminases

## Abstract

**Background:**

Hepatitis in young children with tuberculosis (TB) outside miliary TB is not well described and represents a challenge because of the hepatotoxicity associated with first-line anti-TB treatment.

**Case presentation:**

We report an antibiotic naïve 13-month-old male from Nepal with pulmonary TB and hepatitis, who improved after TB treatment. We also performed a literature review for TB-associated hepatitis in children.

**Conclusions:**

Liver function tests should be considered, when feasible, in infants and young children with pulmonary TB. Testing could help to identify and manage patients with TB-associated hepatic abnormalities, and also to establish a baseline for detection and management of liver injury associated with anti-TB therapy.

## Background

Tuberculosis (TB) disease remains a global health problem, representing the leading cause of death due to a single infectious disease [1, 2]. Young children are at increased risk for disseminated TB, including meningitis, miliary TB, and less frequently, hepatic disease. While the frequency of drug-induced liver injury secondary to anti-TB medications has been reported, the occurrence and management of TB-associated hepatitis in antibiotic naïve children is not well described and represents a challenge because of the possible hepatotoxicity associated with first-line TB therapy [2–5].

We report a 13-month old infant who presented with pulmonary and mediastinal TB in addition to hepatitis. We also reviewed the literature in regards to the occurrence of liver involvement in children with TB. From 2008 to 2019, we searched for the following terms in Pubmed: “hepatic tuberculosis”, “elevated transaminases AND tuberculosis”, “transaminitis AND tuberculosis”, “liver dysfunction AND pulmonary tuberculosis” and, “liver dysfunction AND miliary tuberculosis”. Of the 41 studies identified, five included complete data in pediatric patients and are summarized in Table S1 [4, 6–8], while the remaining 35 studies reported data exclusively in adults, did not specify the anti-TB drugs used or the duration of treatment, patients had underlying liver disease or transaminase levels were not reported (Table S2).

## Case presentation

A 13-month-old healthy Nepali male with history of BCG vaccination was hospitalized for suspicion of pulmonary TB. The patient’s father (index case) was diagnosed with pan-susceptible cavitary TB the previous year, for which he completed an initial six-month course of directly observed therapy with rifampin (RMP), ethambutol (EMB) and moxifloxacin. His clinical course was complicated by interruptions secondary to suspected alcohol-induced liver injury. His wife and the infant, who was six-month of age at that time, were evaluated for latent TB infection. The infant’s initial and 8-week follow-up tuberculosis skin test (TST) were non-reactive, and the chest radiograph was normal. He received isoniazid (INH) for < 1 month because of lack of compliance. Two months after the father completed the treatment, he was hospitalized again with cavitary TB that was still pan-susceptible to first-line TB agents. At this time the infant received another TST that showed 8 mm of induration and a chest-radiograph that showed right middle lobe air-space disease and possible mediastinal lymphadenopathy with deviation of superior trachea. Per maternal history he had non-specific symptoms the month before including mildly decreased appetite, cough, unspecified weight loss, and fatigue. He had remained afebrile and had not receive any medications. On physical examination, he was playful and in no distress, his neurologic exam was normal and he did not have any focal deficits. On auscultation he had bibasilar crackles but good bilateral aeration. His abdominal exam was benign, with no masses or organomegaly.

**Fig. 1:**
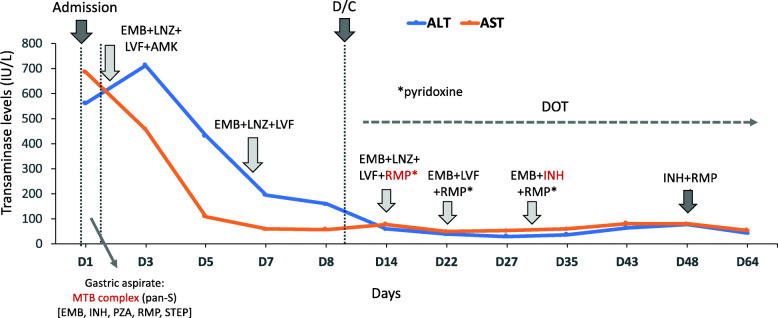
Dynamics of transaminase levels according to initiation of anti-TB treatment. Levels of alanine aminotransferase (ALT, in blue) and aspartate aminotransferase (AST, in orange) are represented in the y axis (IU/L) over time (x axis) according to the initiation of TB treatment and the different anti-TB drugs used. EMB: ethambutol, LNZ: linezolid, LVF: levofloxacin, AMK: amikacin, RMP: rifampin, INH: isoniazid, PZA: pyrazinamide, STEP: streptomycin. MTB: *Mycobacterium tuberculosis*. D/C: discharge. DOT: directly observed therapy

The laboratory evaluation upon admission revealed a strongly positive interferon gamma release assay (QuantiFERON-TB Gold plus; > 0.35 IU/mL ag- nil) and the respiratory PCR panel was positive for RSV. The white blood cell count was within normal limits [15,800/μL (50% lymphocytes)], he was anemic for age (Hg: 9.7 g/dL), and platelets were elevated at 688,000/μL. C-reactive protein (2.4 mg/dL) and erythrocyte sedimentation rate (69 mm/h) were both increased. Electrolytes and coagulation tests were within normal limits. Prior to receiving anti-TB drugs, liver function tests (LFTs) at admission were abnormal (alanine aminotransferase (ALT): 562 IU/L; aspartate aminotransferase (AST): 688 IU/L; Fig. 1).

With concerns for miliary/disseminated TB and to further define the abnormalities identified on the initial chest radiograph, a chest/abdomen CT was performed that showed a right middle lobe opacity and marked enlargement of right hilar, superior mediastinal, and subcarinal lymph nodes. No hepatosplenic lesions were identified. Abdominal ultrasound with doppler was unremarkable and the liver and spleen were normal in size, contour and echotexture. To assess for central nervous system (CNS) involvement, a brain MRI, ophthalmologic examination, and cerebrospinal fluid (CSF) studies were performed and were all normal. AFB staining and PCR for *Mycobacterium tuberculosis* (MTB) in the CSF were both negative.

After one gastric aspirate and two induced sputum samples were obtained, a second line 4-drug TB “liver friendly” regimen was initiated including EMB (20 mg/kg/day, oral); (LVF, 15 mg/kg/day, oral); amikacin (AMK, 15 mg/Kg/day, IV); and linezolid (LNZ, 10 mg/Kg/day, IV). After 48 h, AST was 459 IU/L, ALT 715 IU/L, alkaline phosphatase (ALP) of 184 IU/L and total bilirubin was 0.4 mg/dL. Other causes of hepatitis were assessed for, including CMV, EBV, adenovirus, HIV, enterovirus, and parechovirus, and all resulted negative by blood PCR testing. Mycoplasma was negative by throat PCR. Serologies for hepatitis A, B and C, *Blastomyces, Coccidioides, Histoplasma* and *Aspergillus*, *Histoplasma* urine/serum antigen, and α-1 antitrypsin were negative. The evaluation for mitochondrial liver diseases was negative.

Three days after treatment initiation, LFTs were mildly improved and upon documentation of his father’s MTB pan-susceptibility to first-line agents AMK was discontinued. The patient was discharged receiving oral EMB, LVF and LNZ with directly observed therapy. LFTs 2 weeks after hospital discharge were significantly improved (AST: 79 IU/L, ALT: 60 IU/L; ALP: 173 IU/L), and treatment with RMP (15 mg/Kg/day) was initiated. On follow-up 2 weeks later (25–30 days of therapy), the patient was doing well with normalization of LFTs. At that time LVF was discontinued and INH (10 mg/kg/day, oral) initiated. Gastric aspirate cultures on liquid culture media revealed pan-susceptible MTB complex. The Gen-Probe Amplified *Mycobacterium tuberculosis* Direct (MTD) test (Gen-Probe Incorporated, San Diego, CA), performed on gastric aspirate and CSF samples at the Ohio Department of Health, was negative. He completed 3 more weeks with EMB, RMP and INH followed by INH + RMP for a total of 9 months.

## Discussion and conclusions

Studies suggest that there are five clinical-histologic forms of liver involvement in patients with TB disease: miliary, granulomatous, nodular, ductal and nodal TB [2]. Of all those, the form associated with miliary TB represents the most common in adults and entails widespread liver dissemination and injury [2, 4, 6–9]. The frequency of liver involvement in children due to primary TB is unknown, and although clinical experience suggest that clinically apparent hepatic disease in is rare in pediatric patients, elevated LFTs and ALP before initiating anti-TB treatment may suggest diffuse hepatic disease, which is likely what occurred in our case [2].

The Infectious Diseases Society of America (IDSA) clinical guidelines recommend to screen for LFTs before initiating TB treatment in adults. LFTs then require frequent monitoring only in patients with underlying liver or biliary tract disease, receiving treatment with other hepatotoxic drugs, concomitant HIV infection or previous drug-induced liver injury history [3], due to the hepatotoxicity associated with first-line agents [2, 4, 5, 10]. In children, small studies have reported rates of hepatotoxicity associated with TB treatment that varied from 8 to 40% [11, 12], although overall, the occurrence of drug-induced liver toxicity in children is considerably lower than in adults. Nevertheless, baseline LFTs could serve to monitor its potential development and help to identify hepatic TB, for which early identification and management has been associated with better outcomes [6, 13].

In one of the five studies identified in the literature that included children with hepatic TB (*n* = 4) or liver involvement with miliary TB (*n* = 1), transaminase levels recovered fully after TB treatment (Table S1). Time to recovery was only mentioned in case 5, with full resolution after 6 months of therapy [8]. A study that included adult and pediatric cases (10–87 years old) described a case of mild increase in LFTs in the context of miliary TB, which contrasts with our case, in which transaminase levels were more than 15 times over the upper normal limit.

Abdominal CT is the most sensitive tool to identify liver disease associated with TB, but it is not specific [2, 10]. Hepatobiliary TB could mimic malignancy particularly when a liver mass is associated with obstructive cholestasis [7–9]. In those cases, abdominal MRI could help in the differential diagnosis [8]. Nevertheless, hepatic TB is often underrecognized because initially lesions might be too small [9]. In our patient, the abdominal US and CT were unrevealing, and an abdomen MRI was not performed limiting our ability to rule out microscopic disease. Nevertheless, the definitive diagnosis of hepatic TB requires histologic evaluation, particularly when isolated lesions are present, since no alternative sources are available for microbiology testing [4, 7, 8]. In our case, the patient’s chest CT findings were consistent with pulmonary TB, which allowed us to reliably establish the diagnosis. He also had mild upper respiratory symptoms, likely related to a recent RSV infection and asymptomatic hepatitis likely secondary to TB, since he was not recently exposed to possible hepatotoxic medications at the time of diagnosis, LFTs improved upon initiation of TB treatment, and other common causes of hepatitis were excluded.

The drug regimen recommended for the treatment of TB with liver involvement is similar to pulmonary TB, with variable duration because of the lack of controlled data [2, 4, 8]. According to European, American, and WHO guidelines, 6 months of anti-TB therapy is adequate for miliary TB without CNS involvement [5]. The limited data available suggest that patients with hepatic disease do not require different dosages or classes of anti-TB drugs. In fact, INH and RMP are the preferred agents. In addition, in the majority of low- and middle-income settings where young children receive treatment for disseminated TB with first line standard regimens, LFTs are not evaluated and studies have shown that clinically apparent drug-induced hepatitis is in general rare [13]. In cases of INH intolerance or liver dysfunction not related to TB with ALT less than 3 times the upper limit of normal, the CDC recommends pyrazinamide (PZA), RMP and EMB for 6 months or a 9-month course of initial EMB, INH, and RMP followed by INH and RMP based on susceptibilities, with monitoring of liver enzymes. Liver friendly agents (EMB, fluoroquinolones and streptomycin), which are not without risk for toxicity or treatment response, could be used in cases of severe hepatitis and/or disseminated TB [5].

To our knowledge, this is the first report of an infant with pulmonary TB and asymptomatic hepatitis, and the youngest patient with hepatic TB reported in the literature. This report emphasizes the potential utility of LFT assessment and prompt initiation of TB therapy for treatment of TB disease and rapid improvement of liver dysfunction.

## Supplementary information

**Additional file 1: Table S1.** Characteristics of pediatric patients with elevated liver function tests and TB disease. **Table S2.** Studies in patients with TB and hepatitis.

## Data Availability

All data and materials are provided in the manuscript and supplementary material.

## Abbreviations

ALP: Alkaline phosphatase
ALT: Alanine aminotransferase
AMK: Amikacin
AST: Aspartate aminotransferase
CNS: Central nervous system
CSF: Cerebrospinal fluid
EMB: Ethambutol
IDSA: Infectious Diseases Society of America
INH: Isoniazid
LFTs: Liver function tests
LNZ: Linezolid
LVF: Levofloxacin
MTB: *Mycobacterium tuberculosis*
PZA: Pyrazinamide
RMP: Rifampin
TST: tuberculosis skin test
TB: Tuberculosis
WHO: World Health Organization
