# Supplementary material for: Hepatitis in children with tuberculosis: a case report and review of the literature
Source: BMC Pulm Med. 2020 Jun 16;20:173. doi: 10.1186/s12890-020-01215-6 (PMC7298953; doi:10.1186/s12890-020-01215-6)
Supplement: Supplementary file 1 — Additional file 1: Table S1. Characteristics of pediatric patients with elevated liver function tests and TB disease. Table S2. Studies in patients with TB and hepatitis. [file 12890_2020_1215_MOESM1_ESM.docx]

| Country/Study | Population  N Age (yr) | Clinical  Diagnosis | Co-morbidities | | LFTs  (IU/L) | | Abdominal imaging/  Histopathology | | Treatment  Duration (mo) | | Outcomes | | |
| --- | --- | --- | --- | --- | --- | --- | --- | --- | --- | --- | --- | --- | --- |
| Iran,  [8] | n=1  16 yr | Hepatic TB | - | | AST 55  ALT 37 | | US: nodularities in L lobe of liver. Splenomegaly.  Tuberculous granuloma. | | RMP,INH,PZA, EMB  x2 mo  RMP,INH x4 mo | | Complete resolution | | |
| India,  [7] | n=1  3 yr | Hepatic TB | - | | AST 70  ALT 45  ALP 508 | | US: abscess in R lobe of liver. | | RMP,INH,PZA, EMB  x2 mo  RMP,INH x4 mo | | Complete resolution | | |
| India,  [5] | n=1  20 yr | Hepatic TB | - | | AST 260  ALP 308 | | US: asictes, splenomegaly, nodularity.  CT: granulomatous infiltration.  Tuberculous granuloma. | | Ofloxacin, RMP,EMB  Subsequenty added: INH,PZA  x12 mo | | Complete resolution | | |
| UK,  [3] | n= 52  10-87 yr | Miliary TB | HIV 3/49 (6%)  HBV 3/49 (6%)  HCV 1/47 (2%)  Alcoholism 3 (6%) | | ALT  46  (5-453)  ALP  121  (39-866) | | - | | RMP, INH, PZA, EMB  x12 mo (1.5-28)  (10/52—19%-- pts discontinued within 12 d)  29 (56%) corticosteroids | | Critical care intervention  8 (15%)  1 yr Mortality 2/49 (4%) | | |
| Oman,  [9] | n= 1  19 yr | Hepatic TB | | - | | ALT 457  ALP 302 | | CT: hepatic hilar mass with a central necrosis.  Tuberculous granuloma. | | RMP, INH x6 mo | | Complete resolution |  |

**Table S1. Characteristics of pediatric patients with elevated liver function tests and TB disease.**

Abbreviations: yr, years; mo, months; L, left; R, right; HBV, Hepatitis B virus; HCV, hepatitis C virus; pts, patients; d, days. Liver function test are reported as medians (range). RMP: rifampin, INH: isoniazid, PZA: pyrazinamide, EMB: ethambutol.

**Table S2. Studies in patients with TB and hepatitis**

| Country/  Study | Population  N  Age (yr) | | Clinical  Diagnosis | Co-morbidities | | LFTs  (IU/L)  Median  (range) | Abdominal imaging  Histopathology | Treatment  Duration (mo) | Outcomes |
| --- | --- | --- | --- | --- | --- | --- | --- | --- | --- |
| Spain,  [11] | n=1  4 yr | | Hepatic TB | - | | Elevated transaminases | US and CT: Nodules | -  - | Complete  Resolution |
| Africa,  [12] | n=1  12 yr | | Hepatic TB | - | | ALP 180 | US: nodular hepatomegaly  Tuberculous granuloma | -  - | Complete  Resolution |
| India,  [13] | n=1  14 yr | | Miliary TB | - | | ALP 750 | US and CT: portal thrombosis and cholangitic liver abscesses | RMP, INH, PZA, EMB  Dexamethasone  Duration UK | - |
| New York,  [14] | n=109  16-74 yr | | Miliary TB | 50% alcoholism | | 50% elevated transaminases | Tuberculous granuloma (31/38) | PAS, isonicotinic hydrazide, STEP +/- cycloserine  Duration UK  Prednisone | Mortality 28% (30/109) |
| China,  [15] | n=16  18-56 yr | | Pancreatic TB (16)  Pulmonary TB (7) | - | | 9 pts elevated transaminases | - | RMP,INH,PZA, EMB  or RMP,INH,PZA,STEP  x6 mo  RMP,INH,STEP x10 mo  Surgery (3) | Complete  Resolution |
| France,  [16] | n=20  20-85 yr | | Abdominal TB | Alcoholism (1) | | GGT elevated  (6/21=29%)  AST elevated (11/18=61%) | US (6/20): ascites (4), lymphadenopathy (1), peritoneal abscesses (1)  CT (19/20):  Lymphadenopathy (47%), ascites (47%)  Tuberculous granuloma (18/19) | RMP,INH,PZA, EMB  x6 mo (14%)  >6 mo (67%) | Lost F/U 3  1 deceased |
| Brunei,  [17] | n=5  33-54 yr | Hepatic TB | | - | | ALP  (221-911)  ALT  (130-186)  GGT  (106-303) | US (5/5): Single mass (2/5), multiple masses (2/5), hepatomegaly (3/5)  Tuberculous granuloma (5/5) | - | Complete  Resolution |
| Taiwan,  [18] | n=1  37 yr | Pancreatic TB | | - | | AST 244  ALT 630 | US: dilation of bilateral intrahepatic duct | Treatment UK x6 mo | Complete  Resolution |
| Taiwan,  [19] | n=1  63 yr | Hepatic TB | | Gastric cancer | | ALT 188  AST 124 | US: a solitary mass | - | Mortality |
| Saudi Arabia,  [20] | n=1  49 yr | Hepatic TB | | HCV  Cirrhosis  Liver trasplant  (5 mo before) | | AST 238  ALT 292 | Tuberculous granuloma | PZA,EMB,STEP,  ciprofloxacin  x11 mo | Complete  Resolution |
| Chile,  [21] | n=1  42 yr | Hepatosplenic TB | | Alcoholism | | AST 68  ALP 232  GGT 20 | CT: hypodense hepatic image  Tuberculous granuloma | RMP,INH,PZA, EMB  x7 mo | Complete resolution |
| Morocco,  [22] | n=1  28 yr | Hepatic TB | | - | | AST 77  ALT 96 | US: heterogeneous hypoechoic lesions  CT: multiseptate abscess | RMP,INH,PZA, EMB  Duration UK | Complete resolution |
| USA,  [23] | n=1  52 yr | Hepatic TB | | Diabetes type II  End renal stage disease | | AST 274  ALT 192  ALP 794  GGT 1161 | Tuberculous granuloma | RMP,INH,PZA, EMB x30 d  Moxifloxacin and INH,PZA, EMB  x6 d  RMP,INH,PZA, EMB x36d  Total duration UK  Prednisone (60 mg/d) since d 41, tapered 6 weeks. | Complete resolution |
| New York,  [24] | n=1  81 yr | Hepatic TB | | - | | ALP 113. | US: hypoechoic lesions in the right and left lobe  CT: hypodense lesions  Tuberculous granuloma | RMP,INH,PZA, EMB  x12 mo | Complete resolution |
| Pakistan,  [25] | n=1  30 yr | Hepatic TB | | - | | ALP 296  AST/ALT elevated | US: multiple hypoechoic areas. Few lymph nodes  CT: multiple non-enhancing lesions  Tuberculous granuloma | RMP,INH,PZA, EMB  x12 mo | Complete resolution |
| Sri Lanka,  [26] | n=1  30 yr | Hepatic TB | | - | | ALP 510  AST/ALT elevated | US: low echogenic lesions  CT: a large (10 x 9 cm) solid low density area in the left lobe of the liver  Tuberculous granuloma | STEP,INH, EMB x2 mo INH,EMB x5 mo | Complete resolution |
| India,  [27] | n=1  32 yr | Hepatitis TB | | - | | AST 160  ALT 294  ALP 300 | US: multiple hypoechoic lesions in liver  Tuberculous granuloma | - | Deceased (septic shock) |
| India,  [28] | n=1  37 yr | Hepatic TB | | - | | AST 14  ALT 81 | US: echoes with multiple confluent hypoechoic heterogeneous areas  CT: lymph nodes  Heterogeneous lesions  Tuberculous granuloma | -  - | Complete resolution |
| Turkey,  [29] | n=1  42 yr | Hepatic TB | | - | | AST 31  ALT 24  ALP 155 | MRI: tumor-like mass lesion  Tuberculous granuloma | RMP,INH,PZA, EMB  x12 mo | Complete resolution |
| Ohio,  [30] | n=1  61 yr | Miliary TB | | - | | AST 165  ALT 961 | - | - | - |
| Japan,  [31] | n=1  63 yr | Pulmonary TB | | HBC | | AST 110  ALT 95  ALP 6810 | - | RMP,INH, EMB x4 mo  RMP,INH,PZA, EMB  x 5 mo | Complete resolution |
| India,  [32] | n=1  30 yr | Hepatic TB | | - | | AST 245 | US: ascites. Nodularity liver  CT: nodules in the liver  Tuberculous granuloma | Ofloxacin, RMP,EMB  Subsequenty added: INZ+PZA x6 mo | Complete resolution |
| Morocco,  [33] | n=1  30 yr | Hepatosplenic TB | | - | | AST 3ULN  ALT 4ULN  GGT 3ULN  ALP 2ULN | US and CT: nodules  Tuberculous granuloma | RMP,INH,PZA, EMB  x2 mo  RMP,INH x10 mo | Complete resolution |
| India,  [34] | n=1  35 yr | Hepatic TB | | - | | Elevated ALP | CT: biliary cystadenoma  Tuberculous granuloma | - | Complete resolution |
| China,  [35] | n=1  40 yr | Miliary TB | | - | | AST 24  ALT 13  GGT 126 | US and CT: lumps in the liver. Lymphadenitis | - | Complete resolution |
| California,  [36] | n=1  49 yr | Hepatic and bone marrow TB | | - | | AST 500  ALT 500 | US and CT: hepatomegaly with trace ascites | RMP,INH,PZA, EMB  Duration UK | Complete resolution |
| UK,  [37] | n=1  33 yr | Miliary TB | | - | | ALT 705  ALP 314 | - | RMP,INH,PZA, EMB  x2 mo  Prednisolone 40 mg/d | Deceased |
| Lisbon,  [38] | n=1  42 yr | Hepatic TB | | - | | ALP 496 | US and CT: hepatomegaly and abdominal lymphadenopathy  Tuberculous granuloma | RMP,INH,PZA, EMB  x2 mo  RMP,INH x4 mo | Complete resolution |
| Senegal,  [39] | n=1  48 yr | Hepatosplenic TB | | - | | AST 62  ALT 49  ALP 367 | US: focal lesion metastasic appearance. Hilar lymph nodes. Dilation of intrahepatic bile conduct  CT: mutiple nodules, lymph nodes | RMP,INH, PZA, EMB  x7 d  RMP+EMB+L x4 mo  RMP+EMB x5 mo | Complete resolution |
| California,  [40] | n=1  64 yr | Hepatic TB | | - | | Elevated LFTs | CT: hilar mass with intrahepatic biliary dilatation  Tuberculous granuloma | -  - | Complete resolution |
| South Africa,  [41] | n=20  22-58 yr | Pulmonary TB (85%)  Extrapulmonary TB (15%) | | HIV (100%)  HBV (2)  VZV (1)  Hepatosplenic squistosomiasis (1) | | ALT (33-95)  ALP (322-1043) | US (13/20): hepatomegaly and/or lymph nodes  Tuberculous granuloma | RMP,INH, PZA, EMB  x(1-13) mo | Mortality  (2/20) |
| India,  [42] | n=1  50 yr | Hepatobiliary TB | | **-** | | AST 71-1043  ALT 227-876  ALP 227-862 | US and CT: hepatomegaly. Lesion suggesting cholangiocarcinoma  Tuberculous granuloma | **-**  **-** | Complete resolution |
| India,  [43] | n=1  22 yr | Hepatobiliary TB | | - | | AST 98  ALT 104  ALP 777 | US: hepatomegaly, hypoechoic areas  Tuberculous granuloma | RMP,INH, PZA, EMB  Duration UK | Complete resolution |
| New York,  [44] | n=1  40 yr | Hepatic TB | | - | | AST 471  ALT 132 | CT: cystic mass and lymphadenopathy on the portal | - | - |
| India,  [10] | n=1  50 yr | Hepatobiliary TB | | - | | ALP 1881  AST 33  ALT 26  GGT 111 | US: multiple hypoechoic focal lesions and dilatation of intrahepatic biliary. Lymph nodes  CT: Hypodense lesions  Thrombosis of the portal vein  Tuberculous granuloma | RMP,INH, PZA, EMB  x2 mo  RMP,INH x4 mo | Complete resolution |
| Japan,  [45] | n=1  44 yr | Liver hilar  Tuberculous lymphadenitis | | | - | AST 30  ALT 30  ALP 844  GGT 249 | US: mass at the liver hilium  CT: mass as hypovascular tumor | RMP,INH, PZA, EMB  Duration UK | - |

Abbreviations: yr, years; mo, months; -, unspecified; UK, unknown; PAS, para-aminosalicylic acid; STEP, Streptomycin; pts, patients; GGT, gamma-glutamyltransferase; F/U, follow-up; HCV, Hepatitis C virus; d, days; HBV, Hepatitis B virus; VZV, varicella zoster virus; L, levofloxacin.

.

**REFERENCES:**

1. (2018) Global Tuberculosis Report 2018. Geneva. World Health Organization (WHO). License: CC BY-NC-SA 3.0 IGO. [Online]: https://[www.who.int/tb/publications/global_report/en/](http://www.who.int/tb/publications/global_report/en/).

2. Evans RP, Mourad MM, Dvorkin L, Bramhall SR (2016) Hepatic and Intra-abdominal Tuberculosis: 2016 Update. Current infectious disease reports 18:45

3. Underwood J, Cresswell F, Salam AP, Keeley AJ, Cleland C, John L, Davidson RN (2017) Complications of miliary tuberculosis: low mortality and predictive biomarkers from a UK cohort. BMC infectious diseases 17:295

4. Nahid P, Dorman SE, Alipanah N, Barry PM, Brozek JL, Cattamanchi A, Chaisson LH, et al. (2016) Executive Summary: Official American Thoracic Society/Centers for Disease Control and Prevention/Infectious Diseases Society of America Clinical Practice Guidelines: Treatment of Drug-Susceptible Tuberculosis. Clinical infectious diseases : an official publication of the Infectious Diseases Society of America 63:853-867

5. Shastri M, Kausadikar S, Jariwala J, Dave D, Patell R (2014) Isolated hepatic tuberculosis: An uncommon presentation of a common culprit. The Australasian medical journal 7:247-250

6. American Thoracic S, Cdc, Infectious Diseases Society of A (2003) Treatment of tuberculosis. MMWR Recommendations and reports : Morbidity and mortality weekly report Recommendations and reports 52:1-77

7. Nandan D, Bhatt GC, Dewan V, Yadav TP, Singh S (2013) Isolated tuberculous liver abscess in a 3-year-old immunocompetent child. Paediatrics and international child health 33:187-189

8. Mojtahedzadeh M, Otoukesh S, Shahsafi MR, Tahbaz MO, Rahvari SK, Poorabdollah M, Sajadi MM (2012) Case report: portal hypertension secondary to isolated liver tuberculosis. The American journal of tropical medicine and hygiene 87:162-164

9. Al Umairi R, Al Abri A, Kamona A (2018) Tuberculosis (TB) of the Porta Hepatis Presenting with Obstructive Jaundice Mimicking a Malignant Biliary Tumor: A Case Report and Review of the Literature. Case reports in radiology 2018:5318197

10. Kandasamy S, Govindarajalou R, Chakkalakkoombil SV, Penumadu P (2018) Isolated hepatobiliary tuberculosis: a diagnostic challenge. BMJ case reports 2018

11. Fernandez Fernandez MA, Brea J, Porras Gonzalez A, Croche Santander B, Obando Santaella I (2009) [Tuberculosis of atypical presentation]. Anales de pediatria 71:79-80

12. N'Goran K, Akaffou E, Konan AN, N'Goan-Domoua AM (2011) [Miliary hepatic: an unusual ultrasound presentation of liver tuberculosis discover in an immuno-competent adolescent]. The Pan African medical journal 9:1

13. Patel R, Choksi D, Poddar P, Shah K, Ingle M, Sawant P (2016) Primary Tubercular Liver Abscess Complicated by Tubercular Meningitis in Portal Cavernoma Cholangiopathy. ACG case reports journal 3:e196

14. Gelb AF, Leffler C, Brewin A, Mascatello V, Lyons HA (1973) Miliary tuberculosis. The American review of respiratory disease 108:1327-1333

15. Xia F, Poon RT, Wang SG, Bie P, Huang XQ, Dong JH (2003) Tuberculosis of pancreas and peripancreatic lymph nodes in immunocompetent patients: experience from China. World journal of gastroenterology 9:1361-1364

16. Fillion A, Ortega-Deballon P, Al-Samman S, Briault A, Brigand C, Deguelte S, Germain A, Hansmann Y, Pelascini E, Rabaud C, Chavanet P, Piroth L (2016) Abdominal tuberculosis in a low prevalence country. Medecine et maladies infectieuses 46:140-145

17. Kok KY, Yapp SK (1999) Isolated hepatic tuberculosis: report of five cases and review of the literature. Journal of hepato-biliary-pancreatic surgery 6:195-198

18. Shan YS, Sy ED, Lin PW (2000) Surgical resection of isolated pancreatic tuberculosis presenting as obstructive jaundice. Pancreas 21:100-101

19. Huang WT, Wang CC, Chen WJ, Cheng YF, Eng HL (2003) The nodular form of hepatic tuberculosis: a review with five additional new cases. Journal of clinical pathology 56:835-839

20. Alothman A, Al Abdulkareem A, Al Hemsi B, Issa S, Al Sarraj I, Masoud F (2004) Isolated hepatic tuberculosis in a transplanted liver. Transplant infectious disease : an official journal of the Transplantation Society 6:84-86

21. Velasquez MJ, Szigethi QM, Panace VR, Morales IR, Marquez CS, Pefaur PJ, Mocarquer MA, Salinas CP, Beltran BC (2007) [Hepatic-splenic micobacteriosis, unusual form of probable extrapulmonary tuberculosis. Case report and review]. Revista chilena de infectologia : organo oficial de la Sociedad Chilena de Infectologia 24:59-62

22. Hassani KI, Ousadden A, Ankouz A, Mazaz K, Taleb KA (2010) Isolated liver tuberculosis abscess in a patient without immunodeficiency: A case report. World journal of hepatology 2:354-357

23. Luther VP, Bookstaver PB, Ohl CA (2010) Corticosteroids in the treatment of hepatic tuberculosis: case report and review of the literature. Scandinavian journal of infectious diseases 42:315-317

24. Zayer S, Koberstein B, Pohle T (2011) Hepatic lesions in an elderly woman - cancer isn't always the answer. Zeitschrift fur Gastroenterologie 49:39-41

25. Farooq Sheikh AS, Qureshi IH, Saba K, Bukhari MH (2013) Primary isolated hepatic tuberculosis. Journal of the College of Physicians and Surgeons--Pakistan : JCPSP 23:359-361

26. Mendis Abeysekera WY, Dulantha de Silva WD, Ginige AP, Pragatheswaran P, Hewage SK, Kumara Banagala AS (2013) An isolated tuberculous liver abscess in a non-immunocompromised patient. Journal of the College of Physicians and Surgeons--Pakistan : JCPSP 23:667-669

27. Jain D, Aggarwal HK, Jain P, Pawar S (2014) Primary hepatic tuberculosis presenting as acute liver failure. Oxford medical case reports 2014:153-155

28. Pandey A, Singh RK (2014) Focal liver tuberculosis: a case report. The Indian journal of surgery 76:223-227

29. Turkel Kucukmetin N, Ince U, Cicek B, Akman H, Boztas G, Tozun N (2014) Isolated hepatic tuberculosis: a rare cause of hepatic mass lesions. The Turkish journal of gastroenterology : the official journal of Turkish Society of Gastroenterology 25:110-112

30. Jackson WE, John BV (2014) Cholestatic hepatitis and weight loss in a Vietnamese immigrant. Gastroenterology 147:569-570

31. Saitou Y, Hatazi O, Aonuma H, Ogura S, Yamamoto N, Kobayashi T (2014) Pulmonary tuberculoma in a patient with chronic hepatitis C: a clinical pitfall in the treatment strategy. Internal medicine 53:1669-1674

32. Suthar PP, Bumiya RG, Patel K, Patel AB (2015) Incidental diagnosis of liver tuberculosis in a patient with jaundice. BMJ case reports 2015

33. Jira M, Sekkach Y, Abouzahir A, Amezyane T, Ghafir D (2015) [Hepato-splenic tuberculosis]. Presse medicale 44:258-259

34. Sharma R, Dey AK, Mittal K, Udmale P, Singh U, Mitkar S, Hira P (2015) Hepatic Tuberculosis Mimicking Biliary Cystadenoma: A Radiological Dilemma. Case reports in surgery 2015:390184

35. Liao JR, Zhang D, Wu XL (2015) Pulmonary tuberculosis combined with hepatic tuberculosis: a case report and literature review. The clinical respiratory journal 9:501-505

36. Truong LN, O'Connell R, Oren A (2015) A 49-Year-Old Man with Fever, Erythema Nodosum, and Ankle Swelling. Final Diagnosis: Extrapulmonary tuberculosis with hepatic and bone marrow involvement. Annals of the American Thoracic Society 12:1575-1577

37. Dunphy L, Keating E, Parke T (2016) Miliary tuberculosis in an immunocompetent male with a fatal outcome. BMJ case reports 2016

38. Ribeiro R, Patricio C, Pais da Silva F, Silva PE (2016) Erythema induratum of Bazin and Poncet's arthropathy as epiphenomena of hepatic tuberculosis. BMJ case reports 2016

39. Diallo I, Mbengue A, Gning SB, Amar MA, Ndiaye B, Diop Y, Fall F, PS MB (2016) Hepatosplenic tuberculosis simulating secondary malignant lesions with cholangitis. BMC research notes 9:316

40. Niemiec SM, Vinetz JM, Sicklick JK (2016) Porta Hepatis Mass. JAMA surgery 151:187-188

41. Gounder L, Moodley P, Drain PK, Hickey AJ, Moosa MS (2017) Hepatic tuberculosis in human immunodeficiency virus co-infected adults: a case series of South African adults. BMC infectious diseases 17:115

42. Jain A, Chaturvedi R, Kantharia C, Joshi A, Londhe M, Kekan M (2017) Secondary sclerosing cholangitis in localized hepatobiliary tuberculosis simulating cholangiocarcinoma: a rare case report. BMC gastroenterology 17:126

43. Narayan KS, Kumar M, Padhi S, Jain M, Ashdhir P, Pokharna RK (2018) Tubercular biliary hilar stricture: A rare case report. The Indian journal of tuberculosis 65:266-267

44. Khanijo S, Tandon P (2018) A case of hepatic tuberculosis: A tuberculoma. The Indian journal of tuberculosis 65:91-93

45. Ando N, Iwata K, Yamazaki K, Shimizu S, Sugihara J, Katayama M, Iwata H, Iwashita T, Shimizu M (2019) A case of liver hilar tuberculous lymphadenitis complicated by biliary stricture diagnosed by endoscopic ultrasound-guided fine-needle aspiration. Clinical journal of gastroenterology 12:57-62
